# Metabolomic Profiling Reveals a Role for Androgen in Activating Amino Acid Metabolism and Methylation in Prostate Cancer Cells

**DOI:** 10.1371/journal.pone.0021417

**Published:** 2011-07-18

**Authors:** Nagireddy Putluri, Ali Shojaie, Vihas T. Vasu, Srilatha Nalluri, Shaiju K. Vareed, Vasanta Putluri, Anuradha Vivekanandan-Giri, Jeman Byun, Subramaniam Pennathur, Theodore R. Sana, Steven M. Fischer, Ganesh S. Palapattu, Chad J. Creighton, George Michailidis, Arun Sreekumar

**Affiliations:** 1 Cancer Center, Medical College of Georgia, Augusta, Georgia, United States of America; 2 Department of Biochemistry and Molecular Biology, Medical College of Georgia, Augusta, Georgia, United States of America; 3 Department of Urology, Medical College of Georgia, Augusta, Georgia, United States of America; 4 Department of Surgery, Medical College of Georgia, Augusta, Georgia, United States of America; 5 Department of Statistics, University of Michigan, Ann Arbor, Michigan, United States of America; 6 Division of Nephrology, Department of Internal Medicine, University of Michigan, Ann Arbor, Michigan, United States of America; 7 Metabolomics Laboratory Application Group, Agilent Technologies, Santa Clara, California, United States of America; 8 Department of Urology, The Methodist Hospital, Houston, Texas, Unites States of America; 9 Dan. L. Duncan Cancer Center, Baylor College of Medicine, Houston, Texas, United States of America; Institut de Génomique Fonctionnelle de Lyon, France

## Abstract

Prostate cancer is the second leading cause of cancer related death in American men. Development and progression of clinically localized prostate cancer is highly dependent on androgen signaling. Metastatic tumors are initially responsive to anti-androgen therapy, however become resistant to this regimen upon progression. Genomic and proteomic studies have implicated a role for androgen in regulating metabolic processes in prostate cancer. However, there have been no metabolomic profiling studies conducted thus far that have examined androgen-regulated biochemical processes in prostate cancer. Here, we have used unbiased metabolomic profiling coupled with enrichment-based bioprocess mapping to obtain insights into the biochemical alterations mediated by androgen in prostate cancer cell lines. Our findings indicate that androgen exposure results in elevation of amino acid metabolism and alteration of methylation potential in prostate cancer cells. Further, metabolic phenotyping studies confirm higher flux through pathways associated with amino acid metabolism in prostate cancer cells treated with androgen. These findings provide insight into the potential biochemical processes regulated by androgen signaling in prostate cancer. Clinically, if validated, these pathways could be exploited to develop therapeutic strategies that supplement current androgen ablative treatments while the observed androgen-regulated metabolic signatures could be employed as biomarkers that presage the development of castrate-resistant prostate cancer.

## Introduction

Prostate cancer (PCa) is the commonest solid organ malignancy diagnosed in men in the United States and is the second leading cause of cancer related death in American men [1]. Androgen and the androgen receptor (AR) play an important role in development and progression of PCa, and androgen ablation is one of the main therapeutic options for the treatment of locally advanced or metastatic PCa [2]. Nearly 90% of all patients with metastatic prostate cancer initially respond to castration-induced androgen withdrawal; however, this treatment is often effective for less than 2 years and subsequently progresses to a castration resistant state (castrate resistant prostate cancer, CRPC). CRPC is a lethal disease. [3]. In spite of its clinical resistance to androgen deprivation therapy, CRPC expresses AR [4] and exhibits active androgen signaling through non-traditional activation of the androgen receptor signaling axis [5]. This is best illustrated by the observation of increasing levels of serum prostate specific antigen (PSA), which is an androgen regulated protein and currently used as a marker for biochemical recurrence of the tumor, despite the development of CRPC [6]. It is still debated as to whether this AR activity in CRPC is mediated by high affinity receptors that are sensitive to low levels of circulating androgens or, whether the receptor gains the ability to promiscuously interact with other steroid hormones [4], [7], [8]. The latter is supported by studies that have described a frequent mutation (T877A) within the hormone-binding domain of AR that renders it permissive for binding other steroid hormones and thereby overcoming a specific requirement for androgens [9], [10], [11]. Importantly however, there are no markers currently available, to predict if the tumor will progress into a castrate resistant state. Thus, an understanding of the molecular alterations that result from androgen action in prostate cancer is essential. Multiple groups have interrogated androgen-regulated changes at the transcriptome and proteome levels in PCa cell lines, using gene expression arrays and mass spectrometry [12], [13], [14], [15], [16], [17], [18]. One such seminal study using affymetrix oligonucleotide arrays highlighted the association of androgen signaling in PCa cells with metabolic processes associated with stress responses [19]. Furthermore, androgen-driven proliferation of PCa cells has been shown to involve activation of mammalian target of rapamycin (m-TOR) [20], [21], [22], [23] that by itself is sensitive to metabolic perturbations in the tumor [24], [25]. In spite of this association, there is limited insight into the biochemical alterations induced by androgen action in PCa cells. Using integrative analysis of matched gene expression and proteomic data, earlier we had predicted the activation of amino acid metabolism in androgen-treated LNCaP (androgen sensitive) prostate cancer cells [26]. This expectation was further strengthened by metabolomic profiling of PCa tissues that revealed amino acid metabolism as being one of the hallmarks for early tumor development [27]. Here, we employ mass spectrometry-based profiling of the metabolome of androgen-treated PCa cells, nominate altered metabolites, identify and validate biochemical pathways and evaluate the hormone associated signature in patient-derived tissues. Our results are indicative of androgen-induced elevation of amino acid metabolism and alteration of methylation potential in PCa cells, both of which corroborate our earlier findings using patient-derived localized and metastatic PCa tissues [27].

## Methods

### Cell lines

Prostate cell lines (Immortalized benign – RWPE; androgen-non-responsive – PC3, DU145 and androgen-responsive – VCaP, and LNCaP) were purchased from American Type Culture Collection (ATCC, Manassas, VA). RWPE cells were grown in keratinocyte-SFM media (Invitrogen Corp., Carlsbad, CA) supplemented with 5 ng/ml epidermal growth factor (EGF) and 50 µg/ml bovine pitutary extract (Invitrogen Corp., Carlsbad, CA). VCaP cells were grown in DMEM-Glutamax media (Invitrogen Corp., Carlsbad, CA) supplemented with 10% fetal bovine serum (FBS; Hyclone Labs, Thermo Scientific, Rockford, IL) and 1% penicillin-streptomycin (Hyclone Labs, Thermo Scientific, Rockford, IL). DU145 cells were grown in Minimum Essential Media (MEM) (Invitrogen Corp., Carlsbad, CA) supplemented with 10% FBS (Hyclone Labs, Thermo Scientific, Rockford, IL), 1% penicillin-streptomycin (Hyclone Labs, Thermo Scientific, Rockford, IL) and 1% HEPES (Hyclone Labs, Thermo Scientific, Rockford, IL). PC3 and LNCaP cells, were grown in RPMI-1640 media (Invitrogen Corp., Carlsbad, CA) supplemented with 10% fetal bovine serum (FBS; Hyclone Labs, Thermo Scientific, Rockford, IL) and 1% penicillin-streptomycin (Hyclone Labs, Thermo Scientific, Rockford, IL).. All cells were maintained at 37°C and 5% CO_2_.

### Androgen (R1881) treatment

Equal number of VCaP cells were plated and grown to 60% confluency in DMEM-Glutamax medium as described above. Medium was then replaced for two days by phenol-red free RPMI-1640 medium supplemented with 10% charcoal-stripped FBS and 1% penicillin-streptomycin (Hyclone Labs, Thermo Scientific, Rockford, IL). One set of cells were treated with 10 nM synthetic androgen, methyltrienolone (R1881; Perkin Elmer, Waltham, MA), for either 24 or 48 hrs, whereas vehicle-treated cells (treated with ethanol) served as controls. At the end of the treatment, cells were, trypsinized, pelleted, washed with 50 mM phosphate buffered saline (PBS, pH 7.4) and stored at −140°C until further analyses.

### Sample preparation for mass spectrometry-based examination of metabolome in cell lines

Mass spectrometry-based metabolomics profiling was performed on frozen prostate cell pellets (∼10 million cells). The process of metabolite extraction involved introduction of equimolar mixture of 11 standard compounds dissolved in methanol (Epibrassinolide, [D_3_] Testosterone, [^15^N] Anthranilic acid, Zeatine, Jasmonic acid, Gibberelic acid, [D_4_] Estrone, [^15^N]-Tryptophan, [D_4_] Thymine, [^13^C] Creatinine and [^15^N] Arginine) followed by homogenization of the cells. The homogenate was then subjected to extraction with sequential use of aqueous (chilled water) and organic (chilled methanol and chloroform) solvents in the following ratio 1∶4∶3∶1 (water∶methanol∶chloroform∶water) [28]. The resulting extracts were de-proteinized using a 3 KDa molecular filter (Amicon Ultracel -3K Membrane, Millipore Corporation, Billerica, MA) and the filtrate containing metabolites were dried under vacuum (Genevac EZ-2^plus^, Gardiner, NY). Prior to mass spectrometry analysis, the dried extract was resuspended in identical volume of injection solvent composed of water∶methanol (50∶50) with 0.2% acetic acid and subjected to liquid chromatography (LC) mass spectrometry. As additional controls to monitor the profiling process, an equimolar mixture of 11 standard compounds (Epibrassinolide, [D_3_] Testosterone, [^15^N] Anthranilic acid, Zeatine, Jasmonic acid, Gibberelic acid, [D_4_] Estrone, (^15^N) Tryptophan, [D_4_] Thymine, [^13^C] Creatinine, and [^15^N] Arginine) and a characterized pool of mouse liver (extracted in tandem with cell lines) were analyzed along with the cell line samples. Each of these controls, were included multiple times into the randomization scheme such that samples preparation and analytical variability could be constantly monitored. Furthermore, analysis of each cell line was succeeded by at least two blank runs, to prevent any carryover of metabolites between samples. **Figure S1** illustrates the reproducibility in the profiling process monitored using the standard mixture described above. Notably the CV for the entire profiling process measured using five independent replicates of the mouse liver extract mentioned above was less than 5%.

### Liquid Chromatography/Mass Spectrometry (LC/MS)

The LC/MS portion of the unbiased profiling platform is based on a 1200 SL Rapid resolution LC and a 6520 Quadrapole Time Of Flight (Q-TOF) mass spectrometer (Agilent Technologies, Santa Clara, CA). The samples were independently examined in both positive and negative ionization modes using a dual Electrospray Ionization (ESI) source. Real time mass correction during mass spectrometry was achieved by infusion of a standard mixture of reference ions using an independent 1200 SL Rapid resolution LC isocratic pump equipped with 100∶1 splitter to output a flow rate of 5 µl/min. This reference mixture supplied by the vendor contained ions with *m/z* 121.050873, 922.009798 and 119.03632, 966.000725 for mass correction in the positive (+) and negative (−) ionization modes respectively. A mass range between 50–1000 m/z was employed for the entire unbiased profiling process. The data acquisition during the analysis was controlled using the Mass Hunter workstation data acquisition software. The parameters used during the mass spectrometry included the following: 1) source conditions: capillary voltage, 4000 V (negative mode 3500 V), 2) source temperature was 325°C, 3) drying gas was used at 10 l/min 4), nebulizer pressure was maintained at 45 psig (reference ion nebulizer 10 psig), 5) fragmentor voltage was set at 140, and 6) skimmer voltage was maintained at 65 V. For the entire analysis, ultra high pure nitrogen was used as the nebulizer and collision gas. For collision induced dissociation (CID) experiments, the precursor ion was selected using the quadrupole analyzer set to high resolution mode while the product ions were analyzed by the TOF analyzer. The collision energies in all the experiments were set between 10–40 eV unless otherwise specified. The spectra for the samples analyzed in this study were recorded under identical experimental conditions. LC solvents used for the entire study were purchased from Burdick & Jackson (Muskegon, MI), and used without further purification.

The reverse phase (RP) chromatographic separation on the LC-QTOF employed a gradient using Water (solvent A) and Methanol (MeOH, solvent B) (both solvents were modified by the addition of 0.2% Acetic acid). The binary pump flow rate was 0.6 ml/min with an initial solvent composition of 2% B. The gradient was operated from 2% B to 98% B over a 13 minute period, followed by 98% B for 6 min and 5 min post (sample analysis) time. A column system consisting of a guard column Zorbax SB-C8 (2.1×30 mm, 3.5 um) and analytical column Zorbax SB-Aq (Agilent Technologies, CA) (2.1×50 mm, 1.8 um,) was used for the carrying out the reverse phase separation. The column temperature was maintained at 60°C throughout the chromatographic process. Further, all samples were kept at 4°C prior to their mass spectrometry-based metabolomic analysis. Mass spectral data was acquired in both centroid and profile modes. To monitor any run to run variability, the flow rates and pressure curves for each of the LC-associated pumps were collected and stored.

The LC coupled Triple Quadrupole Mass Spectrometry (QQQ Agilent Technologies, Santa Clara CA) was used for targeted assessment of compounds using Single Reaction Monitoring (SRM) strategy. The operational parameters for this mass spectrometer included 1) source conditions capillary voltage of 3000 V and source temperature of 350°C, 2) drying gas maintained at 10 l/min, 3) nebulizer pressure set at 35 psig and 4) fragmentor voltage set at 70 V. The collision energies used for fragmentation was set at 10–40 eV unless otherwise stated.

The reverse phase (RP) chromatographic separation on the LC-QQQ employed a gradient containing Water (solvent A) and Acetonitrile (ACN, solvent B) (both solvents were modified by the addition of 0.2% Acetic acid and 0.1% formic acid). Chromatographic separation was performed on a Zorbax Eclipse XDB-C18 column (Agilent Technologies, CA) (50×4.6 mm i.d.; 1.8 µm) maintained at 37°C and a flow rate of 0.2 ml/min. The solvent at the start of the gradient was 2% B which was then gradually stepped up to 30% B over 6.5 minutes followed by an increase to 90% B over the next 0.5 minutes, 95% B for an additional 5 minutes and stepped down to 2% B for a period of 8 minutes. This was followed by a post-sample equilibration for an additional 5 minutes. Notably, the column was washed and reconditioned after every 50 injections.

The aqueous normal phase (ANP) chromatographic separation on the LC-QQQ employed a gradient containing Acetonitirle (ACN, solvent A): Water (solvent B) with both solvents modified by the addition of 0.2% Acetic acid and 0.1% formic acid. The flow rate was set at 0.4 ml/min. The initial solvent was 95%A with a gradient from 95%A to 90%A over 3 minute period, 90%A–80%A in 2 min, followed by 80%A–75%A in 1 min, 75%A–55%A in 2 min, 55%A–40%A in 2 min, 40%A–30%A in 2 min, 30%A–20%A in 2 min, 20%A–95%A in 1 min and 95%A for 5 min. The column was then reconditioned back to starting conditions. A Diamond Hydride column (MicroSolv Technology, Eatontown, NJ) (4 um, 100A 2.1×150 mm) was maintained in temperature controlled chamber (37°C) and used for compound separation. During the unbiased and targeted metabolomic profiling process a few compounds are redundantly visualized across multiple platforms. With the understanding that the sensitivity and linearity are vastly different from interface to interface, these redundancies are used as a part of the quality control process. Furthermore, the rigorous quality control adopted in this study involved a chromatographic separation that had less than 0.1 minute variation in retention time of detected compounds between experiments (**Figure S1**). Additionally we also used internal standards containing equimolar mixture of pure compounds as well as a characterized pool of liver specimen for controlling process-associated variation. Both these controls were repeatedly analyzed in tandem with the cell line samples. Also, to ensure uniformity in the profiling process, all the columns and solvents were purchased from a single manufacturer's lot at the start of these experiments.

In addition to the unbiased profiling described above, evaluation of alanine and sarcosine was carried out using isotope dilution gas chromatography-coupled mass spectrometry. Here residual water was removed from the samples by forming an azeotrope with 100 uL of dimethylformamide (DMF), and drying the suspension under vacuum. All of the samples were injected using an on column injector and an Agilent 6890N gas chromatograph equipped with a 15-m DB-5 capillary column (inner diameter, 0.2 mm; film thickness, 0.33 micron; J & W Scientific Folsom, CA) interfaced with an Agilent 5975 MSD mass detector. The *t*-butyl dimethylsilyl derivatives of sarcosine were quantified by selected ion monitoring (SIM), using isotope dilution electron-impact ionization GC/MS. The levels of alanine and sarcosine that eluted at 3.8 and 4.07 minutes respectively, were quantified using their respective ratio between the ion of *m*/*z* 232 derived from native metabolite ([M-O-*t*-butyl-dimethylsilyl]^−^) and the ions of *m*/*z* 233 and 235 respectively for alanine and sarcosine, derived from the isotopically labeled deuteriated internal standard [^2^H_3_] for sarcosine. The limit of detection (signal/noise>10) was ∼0.1 picomole for sarcosine using isotope dilution GC/MS.

### Metabolomic Phenotyping Microarrays

The metabolic phenotyping was performed using 96-well plates containing various metabolites as sole nutrient substrates. The nutrient containing plates were obtained from Biolog Inc (Hayward, CA) and the assay was performed as per the manufacturer's instruction. A total of 88 sugars, 5 nucleotides and 29 amino acids were examined as substrates on two 96-well plates, labeled M1 and M2 by the manufacturer. Essentially, the assay measures the utilization of these metabolites by the growing cells as function of NADH flux which is in turn measured by the extent of reduction of tetrazolium dye. The latter is quantified by spectrophotometry at 590 nm while any non-specific background activity is assessed at 790 nm. For the metabolic phenotyping studies, VCaP cells were starved for 96 h and seeded into the wells of the 96-well plate at a density of 20,000 cells/well. Notably, the VCaP cells were either treated with 10 nM R1881 or vehicle (ethanol) at the time of seeding. The cells were then allowed to grow for 24 h at 37 C at 5% CO_2_. Following 24 h incubation, NADH flux was measured by monitoring the optical density at 590 nm using a spectrophotometer. Simultaneously, readings at 790 nm were also taken to evaluate background activity. The first reading was taken at 24 h after androgen addition and subsequent readings were taken at 1 h intervals up to 30^th^ hour, followed by three additional readings at 34^th^, 42^nd^ and 48^th^ hrs post-androgen addition. The data was tabulated in an excel sheet and analyzed as described under Statistical Methods.

### Metabolomic Libraries

Metlin library (Agilent, Santa Clara, CA) was used to search the mass spectral data. The library was created using approximately 1000 commercially available compounds whose retention time was defined using the RP and ANP chromatographic methods described above. Additionally, the mass and fragment ion information for each of these standard compounds were also obtained in both positive and negative ionization modes and included in the Metlin library.

### Statistical Analysis

Metabolites with more than 60% missing values were removed from the analysis. The metabolic data is left censored due to thresholding of the mass spectrometer data. To account for differences in missingness patterns across different classes, distribution of proportion of missing values in androgen responsive (ARD), androgen non-responsive (ARI) and benign (Ben) samples were examined and compounds with more than 85% missing values in each group and less than 60% missing values overall were considered to have biological missingness. Androgen treated and control samples were treated similarly. Missing metabolite measurements for these metabolites were replaced (imputed) with the detection level (2000). The missing measures in the remainder of metabolites were imputed using the nearest neighbor algorithm with k = 5 using the R-package pamr. Imputed data were then log2 transformed. Normalization for Q-TOF data was done using quantile normalization per samples using the limma R-package. QQQ samples were normalized by median centering and IQR scaling.

Heatmaps, boxplots and Venn diagrams were drawn using R-packages gplot, stats and limma, respectively. Data obtained from different platforms were then scaled per compound and compared across samples. The samples for duplicated compounds were combined by averaging over samples resulted from multiple occurrences of the same compounds. Hierarchical clustering was performed using complete linkage with Pearson's correlation. A two-sided t-test was used to assess the association of each metabolite with the androgen responsiveness status in a two-sample test. The resulting p-values were then adjusted for multiple testing using FDR with q* = 0.2, by calculating the q-values using the R-package fdrtool [29].

Data from metabolic phenotyping assays were first corrected for background signal, obtained from readings of empty wells. The data were then adjusted by subtracting average values of three negative control wells from all other samples at each time point. The effect of androgen treatment on prostate cancer cells grown in amino acid and sugar plates in comparison to control cells were compared using heatmaps and boxplots. In addition, gene set analysis (R-package GSA [30]) was used to assess the overall enrichment of amino acid and sugar pathways, in the respective plates, at each time point.

## Results

### Metabolomic profiling of prostate-derived cell lines

**Figure 1:**
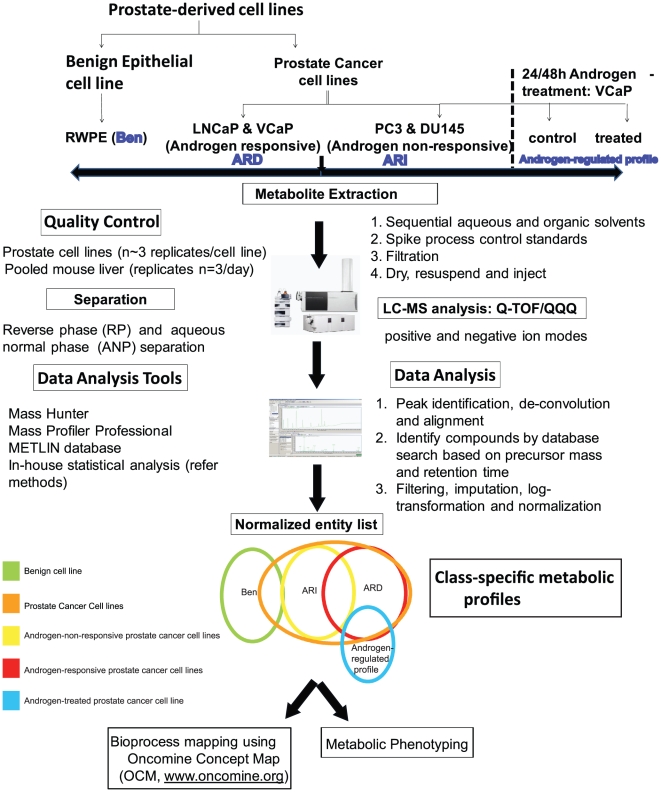
Metabolomic profiling of prostate cell lines. Illustration of the various steps involved in metabolomic profiling of prostate cell lines. The major steps involved metabolite extraction and separation, mass spectrometry-based detection, spectral analysis, data normalization, delineation of class-specific metabolites and altered pathways and their functional characterization. The variation in sample extraction, separation and mass spectrometry were controlled using spiked standards and assessed using various quality control parameters (**Figure S1**).

In an effort to profile the androgen-regulated metabolome in prostate cancer, we used liquid chromatography coupled with mass spectrometry to interrogate the relative levels of metabolites across prostate-derived cell lines (Immortalized benign – RWPE; androgen-non-responsive – PC3 and DU145 and androgen-responsive – VCaP, and LNCaP). In addition to delineating prostate cancer-specific (PCa) metabolic profiles, we also examined metabolic changes in androgen-responsive (ARD) vs non-responsive cells (ARI), as well as those directly regulated by androgen in VCaP cells. As outlined in **Figure 1**, the unbiased metabolomic profiling platform used in this study consisted of both reverse phase and aqueous normal phase chromatography coupled with 1) electrospray ionization (ESI) in the positive ion mode (+) and 2) ESI in the negative ion mode (−). In addition, a targeted assessment of 57 compounds was performed using Single Reaction Monitoring (SRM) on a triple quadrupole mass spectrometer operated in (+) and (−) ion modes respectively (**Figure 1**). The cell line-derived mass spectrometry profiles were subjected to pre-processing that involved data filtering, imputation, logarithmic transformation and normalization as illustrated in **Figure 1**. The normalized data was then interrogated for metabolites that distinguish 1) benign prostate cell line (Ben) from prostate cancer cell lines (PCa) 2) androgen responsive prostate cancer cells (ARD) from androgen independent cells (ARI) and 3) androgen-treated prostate cancer cells from untreated controls. Further, the metabolites that distinguished ARD and ARI cell lines were evaluated in localized and metastatic tissues, using the tissue-derived metabolomic profiles that were earlier published by our group [27]. The class-specific metabolic signatures were subjected to enrichment-based bioprocess mapping followed by validation using in vitro metabolic phenotyping experiments.

**Figure 2:**
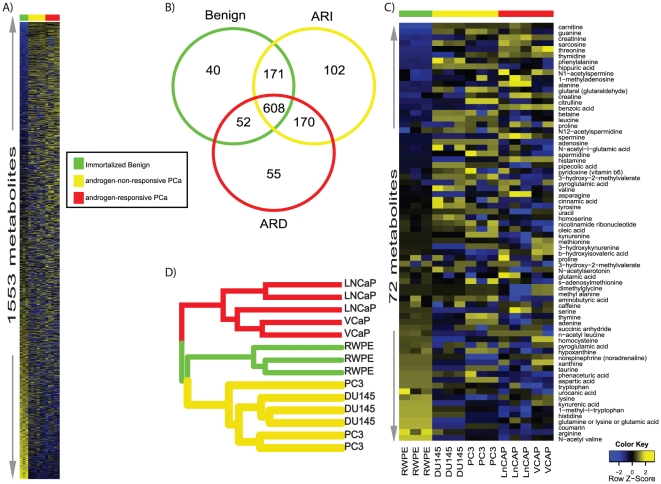
Metabolome of prostate cancer cell lines. **A**) Heat map representation of hierarchical clustering of 1,553 metabolites across 5 prostate cell lines. Sample classes are indicated by the colored bars [benign = green, androgen non-responsive PCa (ARI): yellow and androgen responsive PCa (ARD): red bar]. Columns represent individual cell lines and rows refer to distinct metabolites. Shades of yellow represent elevation of a metabolite and shades of blue represent decrease of a metabolite relative to the median metabolite levels (see color scale). **B**) Venn diagram representing the distribution of 1,553 metabolites measured across benign (RWPE), ARI (DU145 and PC3) and ARD (LNCaP and VCaP) cell lines using liquid chromatography coupled mass spectrometry. **C**) same as in (**A**), but for 72 named compounds **D**) Dendrogram representing hierarchical clustering of the prostate-related cell lines described in B, using compounds with significant differential expression.

A total of 1553 compounds were detected across the six prostate cell lines using the different mass spectrometry methods used in this study (see above, **Figure 2A**). Of these, 40 compounds were detected only in Ben while 102 and 55 compounds respectively were seen in ARI and ARD cell lines (**Figure 2B**). Notably, this compendium contained 72 named metabolites (**Figure 2C**). A hierarchical clustering profile based on compounds with significant differential expression perfectly delineates the prostate cell lines (**Figure 2D**).

### Specific metabolic profiles distinguish Ben from PCa and ARD from ARI

**Figure 3:**
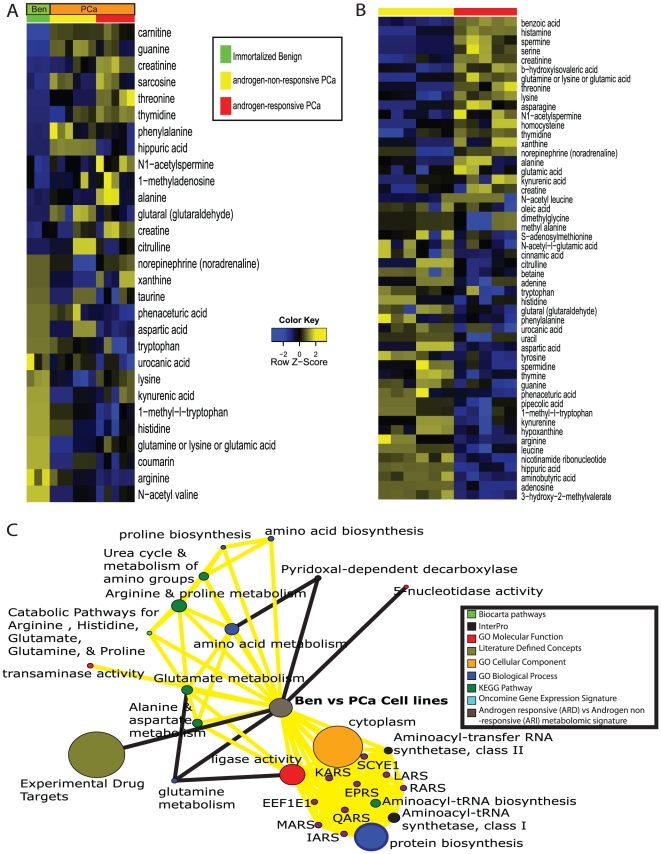
Metabolic alterations in prostate cancer cell lines. **A**) Heat map showing 29 named differential metabolites in prostate cancer cells relative to benign cell line (*p*<0.05, FDR = 20%), derived using a *t*-test coupled with permutations (*n* = 1,000) to determine the *p*-values for comparing the groups. The heat map was generated after log scaling and quantile normalization of the data. The color scheme is the same as in **Fig. 2A**. **B**) same as in (**A**) but for 52 named differential metabolites between ARI (yellow bar) and ARD (red bar) prostate cancer cells. **C**) Network view of the molecular concept analysis for the metabolomic profiles of our “altered in PCa cell line signature” (grey node). Each node represents a molecular concept or a set of biologically related genes. The node size is proportional to the number of genes in the concept. Each edge represents a statistically significant enrichment (FDR *q*-value<0.2). Enriched concepts describing “amino acid metabolism” are indicated by yellow bridges.

To delineate the metabolomic alterations between Ben and PCa cell lines, we used a 2-sample *t*-test. A total of 674/1553 compounds were differential across these two classes after multiple comparison adjustment at a false discovery rate (FDR) of 20%. Included in this list were 29 named metabolites whose relative levels are shown in **Figure 3A**. This altered metabolomic signature in PCa cell lines contained elevated levels of amino acids like sarcosine, threonine, phenylalanine and alanine as well as higher levels of compounds associated with nitrogen metabolism like creatine, creatinine, citrulline and N-acetyl spermine. On the other hand, levels of metabolites belonging to tryptophan metabolism namely tryptophan, 1-methyl tryptophan and kyneuric acid were reduced in PCa compared to Ben. Similarly, 913/1553 compounds were altered between ARI and ARD PCa cell lines (**Figure 3B**). The altered list included 52 named metabolites among which levels of spermine, N1-acetylspermine and amino acids like serine, threonine, lysine, homocysteine, asparagine, alanine, glutamic acid etc, were elevated in ARD (**Figure 3B**). On the contrary, levels of S-adenosylmethione (SAM, methylation currency of the cell) were reduced in ARD cells with a concomitant increase in levels of its break down product, homocysteine (**Figure 3B**). This is suggestive of increased methylation activity in prostate cancer and is in agreement with our earlier findings on advanced prostate cancer tissues [27].

### Integrative Molecular Concept Modeling of PCa-derived metabolome

Upon delineating the metabolomic patterns for Ben, ARD and ARI prostate cell lines, we subsequently pursued evaluation of these changes in the context of biochemical pathways and delineation of altered biochemical processes during prostate cancer development and progression keeping in context its response to androgen. To delineate the overall biological processes that are altered in prostate cancer cell lines, as well as in androgen-responsive prostate cancer cells, the class-specific coordinated expression profiles of metabolites described above were examined using Oncomine Concept Maps (OCM, www.oncomine.org) [31], [32]. Here a ‘molecular concept’ is defined as any set of molecular components that are related in some biologically meaningful way. For this analysis, we used molecular concepts of two general types: (1) gene and protein annotations from external databases, and (2) computationally-derived regulatory networks. The external annotation included chromosomal locations, protein domains and families, molecular functions, cellular localizations, biological processes, signaling and metabolic pathways, protein-protein interaction networks, protein complexes, and gene expression signatures. The regulatory networks were derived by scanning human promoters for known transcription factor motifs and by comparative genomics analyses that identified conserved promoter and 3′UTR elements. In total, data from 13 databases and 335 high-throughput datasets were collected and analyzed. Prior to the enrichment analysis, significant metabolites were assigned to their compound IDs using Kyoto Encyclopedia of Genes and Genomes (KEGG, www.genome.jp/kegg). These were again converted into their corresponding enzyme-gene IDs (EGID) using KEGG, with the premise that metabolites are functional end products of activity of their corresponding enzymes. The resulting EGID's obtained from the class-specific metabolome were then used to select molecular concepts of potential interest where enrichment analysis was carried out against a null set containing a compendium of all EGIDs found in KEGG. The molecular concepts that were selected for were significant at an FDR threshold of 20%. Such an approach allowed us to systematically link metabolomic signatures to bioprocesses associated with prostate cancer and regulated by androgen action. A subset of selected molecular concepts enriched by the PCa-specific metabolome is displayed in **Figure 3C** and listed along with the corresponding FDR adjusted *q*-values in supplementary **Table S1**. Further, the list of all the concepts that were enriched by the PCa-specific metabolome, are listed in supplementary **Table S2**.

Notably, the PCa-specific metabolic profiles enriched for multiple interconnected concepts that described amino acid metabolism, similar to those observed earlier upon integrative analysis of androgen-regulated transcriptomic and proteomic signatures [33] (**Figure 3C**, yellow bridges). These concepts described enriched metabolic activity for proline, arginine, alanine, aspartate, glutamate, glutamine and histidine as well as transaminase activity and mobilization of the resulting nitrogen via the urea cycle. A second set of concepts described utilization of these amino acids for protein synthesis via enrichment of aminoacyl-tRNA's and translational machinery. Interestingly, the metabolomic signature that delineated ARD from ARI also enriched for similar concepts describing elevated amino acid metabolism in androgen responsive cells (**Figure S2**). In addition, the PCa-specific metabolic signature also enriched for nucleotidase and ligase activity, suggestive of cell proliferation (**Figure 3C**).

### Delineation of androgen-regulated metabolites in hormone-responsive prostate cancer

Our results on prostate cancer cell lines, described above and clinically localized tissues reported earlier [27], suggest a role for androgen in preferentially elevating the ability of tumors to metabolize amino acids. To confirm this and to delineate additional metabolic niches affected by androgens, we carried out an unbiased metabolic profiling of androgen-treated VCaP cells. Specifically, these cells were treated with 10 nM R1881 for 24 or 48 h and their metabolome was examined against vehicle (ethanol)-treated cells as controls, using methodology described in **Figure 1**.

**Figure 4:**
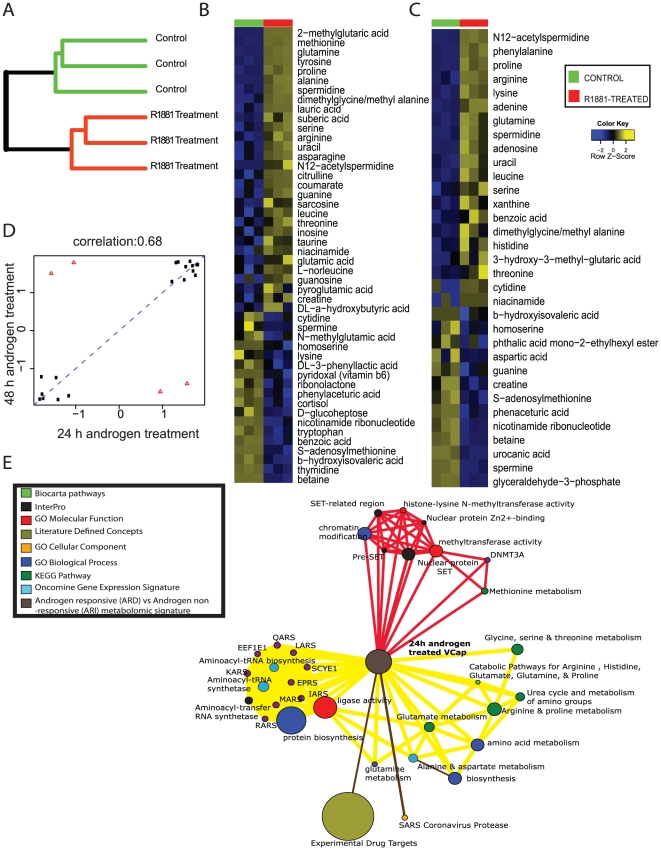
Metabolic alterations upon androgen-treatment of prostate cancer cells. **A**) Dendrogram representing unsupervised hierarchical clustering of the androgen-treated and control cells using their total metabolic profiles. **B**) Heat map showing 48 named differential metabolites that are altered in VCaP prostate cancer cells 24 h after treatment with 10 nM synthetic androgen (R1881) compared to untreated controls, (*p*<0.05, FDR = 20%), derived using a *t*-test coupled with permutations (*n* = 1,000) to determine the *p*-values for comparing the groups. The heat map was generated after log scaling and quantile normalization of the data. The color scheme is the same as in **Fig. 2A**. **C**) same as in (**B**) but for metabolic alterations after 48 h of androgen-treatment **D**) Correlation plot for metabolites altered at 24 h and 48 h post-androgen treatment in VCaP prostate cancer cells **E**) Network view of the molecular concept analysis for the metabolomic profiles of our “Androgen-induced metabolomic signature” (grey node). Each node represents a molecular concept or a set of biologically related genes. The node size is proportional to the number of genes in the concept. Each edge represents a statistically significant enrichment (FDR *q*-value<0.2). Enriched concepts describing “amino acid metabolism” and “methylation potential” are indicated by yellow and red bridges respectively.

The metabolic compendia detected in 24 and 48 h androgen-treated cells contained a total of 1024 and 1146 compounds respectively, which completely distinguished androgen-treated cells from controls in an unsupervised manner (**Figure 4A**). Included in this time course compendia were 69 and 56 named metabolites that were identified at the two time points respectively (**Figure 4B, C**). Interestingly about 70% of the compounds altered by androgen showed a consistent pattern of change at 24 and 48 h time points (**Figure 4D**). Furthermore, similar to the results with PCa cell lines, the list of significant metabolites elevated by androgen-treatment predominantly contained amino acids and components of nitrogen metabolism pathway (**Figure 4 B,C**). Also, similar to our earlier findings with ARD cell lines, androgen treatment of VCaP lowered the SAM levels in these cells with a corresponding increase in levels of methylated metabolites like N-methyl glycine (sarcosine), 2-methylglutaric acid, dimethylglycine, methyl valine etc (**Figure 4B**). This suggests a possible role for androgen in altering methylation potential of prostate cancer cells which, as we reported earlier using tissues [**27**], is a critical component for tumor progression.

To move beyond individual metabolic changes and understand effect of androgens on the biochemical processes as a whole, we performed the enrichment analysis of androgen-regulated metabolic signature obtained 24 h after hormone treatment. A subset of selected molecular concepts enriched by this signature, are displayed in **Figure 4D** and, the list of all the concepts that were enriched are listed in supplementary **Table S3** along with their corresponding FDR adjusted *q*-values.

Notably, as expected, the OCM analysis of androgen-regulated metabolome revealed enrichment of amino acid metabolism similar to our results with ARD cells, as well as portrayed multiple concepts that reconfirmed androgen-mediated alteration of methylation cascade (**Figure 4D**). Interestingly, while enrichment of amino acid metabolism by androgen action echoed the bioprocesses seen in clinically localized prostate cancer tissues, the change in methylation potential was similar to findings seen in metastatic tumors [27]. This suggests a potential dual role for androgen, one wherein it enhances tumors ability to preferentially utilize amino acids early on during their development, and a second, where it potentiates change in the methylation potential that is essential for tumor progression.

### Metabolic phenotype analysis confirms androgen-induced elevation of amino acid metabolism in prostate cancer cells

The metabolomic profiling results described above highlight androgen-induced dependence of prostate cancer cells on amino acids for their development. To confirm this, we tested the ability these cells to metabolize various nutrient substrates that included sugars, nucleotides and amino acids. In essence, this so called Metabolic Phenotyping Array (Biolog, Inc., Hayward, CA), measured the flux of NADH generated as a result of utilization of metabolic substrates by the actively proliferating cancer cells.

**Figure 5:**
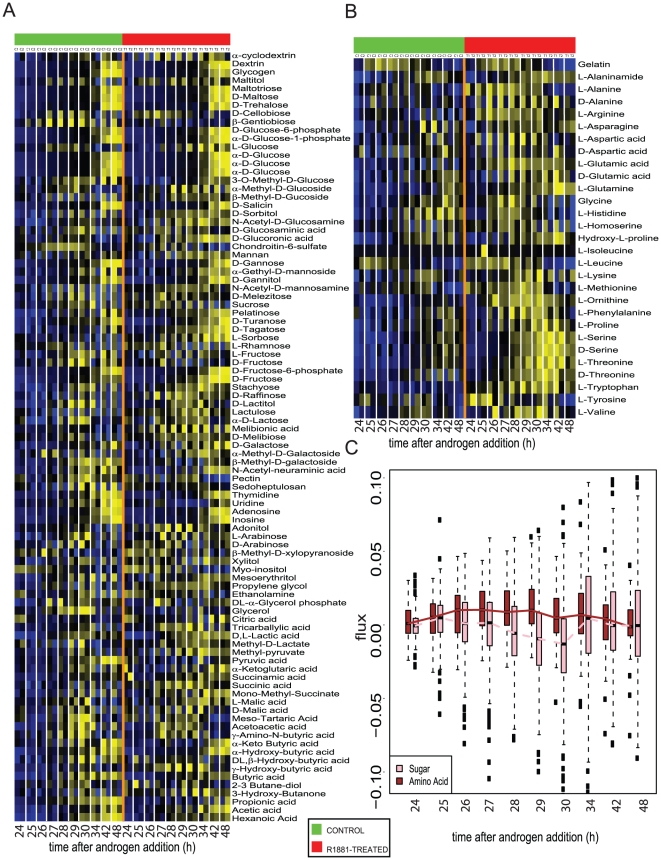
Measurement of metabolic flux in prostate cancer cells upon androgen-treatment. Heat map showing extent of utilization 86 carbohydrate and nucleotide substrates by prostate cancer cells after 24 h of androgen-treatment. Essentially, 10 nM R1881 (treated for 24 h) or untreated VCaP cells were examined in replicates for their ability to utilize the various nutrient substrates (denoted as pathway activity on Y-axis) at different time points (plotted on X-axis) post-androgen-treatment. The ability to metabolize the nutrients was assessed by calculating the flux of NADH generated which was measured by reading the optical density (OD) at 590 nm. The heat map was generated after background subtraction, log scaling and quantile normalization of the data. Shades of yellow indicate increased activity while shades of blue indicate reduced activity (refer color scale). **B**) same as in (**A**), but for utilization of 29 amino acids. **C**) represents the box plot showing the median flux through the sugar and nucleotide (pink) as well as amino acid (brown) pathways in prostate cancer cells at different time points post-androgen treatment. For each boxplot, the median value is represented by the central, horizontal line; the upper (75%) and lower (25%) quartiles are represented by the upper and lower borders of the box. The upper and lower vertical lines extending from the box represent 1.5 times the inter-quartile range from the upper and lower quartiles.

As shown in **Figure 5A** and plotted in **Figure 5C**, upon androgen treatment, prostate cancer cells did not show any time-dependent alteration in metabolism of sugar and nucleotide substrates compared to untreated controls. However, starting at 24 h after androgen addition, these cells exhibit higher metabolism of amino acids compared to untreated cells (**Figures 5 B, C**). This elevated trend in amino acid metabolism in androgen-treated cells was significant at 27 hrs post hormone treatment and persisted till the 30 h time point (GSA *p*-values 0.0001 for all three time points, See Statistical Methods for details) (**Figures 5 B, C**). This increased activity in amino acid pathway correlates well with our cell line-derived metabolic profiles that show androgen driven elevation in levels of these metabolites in prostate cancer. Further, this androgen-mediated increase in amino acid metabolism in prostate cancer cells could potentially result in efficient utilization of methionine (**Figure 5B**, methionine) that could in turn generate higher levels of SAM, thus altering the methylation potential of the cell.

### Comparison of cell line-derived androgen-associated metabolomic signature with tissue-derived metabolome

In this study, insights into the androgen-regulated signature was obtained by first comparing the metabolome of hormone independent cell lines (also termed ARI namely DU145 and PC3) with metabolic profiles of androgen responsive cells (also termed ARD namely VCaP and LNCaP). Even though the androgen response for these cell types have been well documented, these cells exhibit differences in their molecular composition Thus the metabolomic signature that distinguishes the ARD and ARI cell lines could be confounded by differences in the molecular make up of these cells in addition to the difference in their response to androgen. To guard against the former we also profiled the metabolome of VCaP cells treated with synthetic androgen. A concordance analysis of the two metabolic signatures (ARI vs ARD profile and metabolome altered by androgen addition) was performed to home in on metabolites potentially regulated by androgen. Further to determine their relevance in the context of PCa development and progression, a similar meta-analysis was carried out between the androgen responsive metabolome and tissue-derived metabolomics signature, published earlier [27]. The latter comprised of a compendia of metabolites that were detected in organ-confined PCa (n = 12) compared to benign adjacent tissue (n = 16) and in metastatic disease (n = 14) compared to localized PCa [27]. Thus in essence this meta-analysis approach examined metabolomics fingerprints that distinguish ARI vs ARD cells with metabolomics profiles of 1) 24 h androgen-treated cells 2) 48 h androgen-treated cells 3) localized PCa and 4) metastatic disease.

A total of 43/52 metabolites present in at least one of the four datasets described above, were selected for this meta-analysis (**Figure S3**). Initially, we compared the metabolomic profiles that distinguished ARI from ARD with those associated with androgen-treated VCaP cells (24 and 48 h). In this comparison, 26/43 metabolites were concordant. Among others, the latter included amino acids like asparagine, aspartic acid, glutamine, glutamic acid, serine, threonine, histidine, alanine and tryptophan, as well as components of nitrogen metabolism like creatine, creatinine and acetylated-polyamine. Further this list also included SAM and its metabolic product homocysteine.

Similarly, a total of 24/43 metabolites from the ARD vs ARI signature showed a similar pattern of change in the metabolome associated with organ-confined PCa and metastatic disease. The compounds that were concordant in this comparison included amino acids, SAM as well as components of tryptophan breakdown like kynurenine, kynurenic acid and metabolites like histamine, spermine and spermidine. Importantly, a total of 12 metabolites that distinguished PCa cell line based on their response to androgen had similar pattern of expression in both androgen-treated VCaP cells and patient-derived tissues. These included SAM, nicotinamide adenine dinucleotide (NAD), asparagine, serine, histidine, tryptophan, homocysteine, creatinine, hypoxanthine, aspartic acid, tyrosine and lysine. OCM-based enrichment analysis (using all the named metabolites detected in ARD and ARI cell lines (n = 72), as the null set) of this 12 metabolite signature revealed multiple concepts that portray methionine metabolism, SAM activity and methylation (FDR<20%, **Figure S4**, refer to **Table S4** for a list of all concepts enriched by the 12-metabolite signature with associated *p*-values and *q*-values). Notably, these enriched concepts are similar to the ones seen in metastatic PCa [27] and support our notion that androgen action in PCa could alter methylation potential and thus aid in progression of the tumor. Further study is warranted to verify the role of these 12 metabolites in PCa.

## Discussion

To delineate the biochemical processes altered by androgen action, we adopted a strategy of global mass spectrometry-based unbiased profiling of metabolome in prostate cancer cell lines. The profiling data was then used to delineate altered bioprocesses using bioinformatics-based pathway mapping followed by pathway functional validation using metabolic phenotyping microarray assays. Overall, we profiled both benign and prostate cancer cell lines with the latter including both androgen responsive and non-responsive cells. Although multiple groups have interrogated androgen-regulated changes at the transcriptome and proteome levels in these cell lines [12], [13], [14], [15], [16], [17], [18], the androgen-induced metabolomic alterations in these cells have not been reported. The need to define this stems from our earlier findings on metabolomic profiles of prostate cancer tissues that revealed alterations in amino acid metabolism and methylation potential as hallmarks for indolent and advanced tumors respectively [27]. However, from that study it was not clear if these alterations were influenced by androgens, a critical regulator for prostate cancer development and progression.

The unbiased metabolomic assessments made in this study employ highly reproducible chemo-centric chromatographic separations of metabolites coupled to their detection using Q-TOF/QQQ mass spectrometers. The overall process variation in the profiling process was below 5% as monitored using multiple sets of standards and characterized tissue extracts. Using such a robust strategy, the study detected over 1000 compounds in prostate-derived cell lines both in presence and absence of androgen treatment. Importantly, these metabolic profiles could delineate the cell lines into different classes in an unsupervised hierarchical clustering, suggestive of existence of class-specific metabolic niches. Accordingly, comparison of metabolic profiles between PCa and Ben cell lines revealed altered levels of multiple amino acids. Included in this list was sarcosine that was elevated in PCa compared to benign, a finding that was concordant with our earlier profiling study on prostate tissues [27]. Furthermore, components of nitrogen metabolism, namely creatinine, citrulline, creatinine and N1-acetyl spermine were also elevated, indicative of increased amino acid utilization. Notably, this amino acid addiction was reflected in the bioprocess enrichment wherein most of the concepts enriched by the PCa-specific metabolome highlighted altered amino acid metabolism. Further, this pronounced alteration of amino acid metabolism was also observed in androgen responsive prostate cancer cells compared to their hormone non-responsive counterparts. The latter motivated us to examine the role of androgen in instilling amino acid dependency in prostate cancer cells. As expected, both the metabolic data and bioprocess enrichment analysis confirmed elevation of amino acid metabolic processes in prostate cancer. In addition, these also revealed altered methylation potential in androgen-treated prostate cancer cells. This result was intriguing in the context of our earlier findings with the metabolome of metastatic prostate cancer that echoed elevated methylation potential [27]. Furthermore, methylation-induced epigenetic changes are known to be causal in cancer progression [34], [35], [36], [37], [38], [39], [40]. Also, androgen receptor expression in prostate cancer has long been known to be regulated, in part, by methylation [41], [42], [43], [44], [45]. In light of these studies, our results indicative of a role for androgen in stimulating methylation potential is interesting. This, we believe, could be a consequence of increased amino acid utilization by prostate cancer cells, potentially under the influence of androgen. We confirmed this using metabolic phenotyping microarrays that revealed a time-dependent increase in utilization of amino acids by prostate cancer cells upon androgen treatment without any appreciable change in the steady state activity of sugar and nucleotide metabolic pathways. This increase in amino acid metabolism encompasses efficient utilization of methionine that can generate higher levels of SAM, the methylation currency of the cell. Interestingly, OCM analysis of the metabolic signature that was concordant between cell lines and prostate cancer tissues described the enrichment of methionine metabolism and SAM activity, supporting the above premise. Furthermore, higher levels of SAM could promote methylation as evidenced by elevated levels of methylated metabolites like N-methylglycine, a.k.a. sarcosine, 2-methyl glutaric acid, dimethylglycine, methyl alanine etc in prostate cancer cells upon androgen treatment, as well as increased expression of methyltransferases like EZH2, in metastatic disease. From the clinical perspective, this finding of a role for androgen in elevating cancer cell methylation potential could have implications for the development of therapeutic agents [46]. This premise is supported by in vitro studies using 5-Aza-2′-deoxycytidine that have shown a decrease in prostate cacner cell clonogenicity [47].

In summary, here we describe the androgen regulated metabolome in prostate cancer cell lines (metabolomic alterations induced by androgen) and compare these with prostate-derived tissues that included benign, localized tumors and metastatic disease. Our results indicate a potential role for androgen in regulating prostate cancer cell amino acid metabolism as well as methylation potential.

## Supporting Information

Figure S1 Reproducibility of metabolomic profiling platform used in the discovery phase. Chromatographic reproducibility of a mixture of 12 metabolite standards over five technical replicates anaylyzed on the Q-TOF using positive ionization.
(PDF)

Figure S2 Network view of the molecular concept analysis for the metabolomic profiles of our “metabolic profiles that are altered in ARD vs ARI cell lines” (grey node). Each node represents a molecular concept or a set of biologically related genes. The node size is proportional to the number of genes in the concept. Each edge represents a statistically significant enrichment (FDR q-value<0.2). Enriched concepts describing “amino acid metabolism” are indicated by yellow bridges.
(PDF)

Figure S3 Table describing the concordance of metabolomic signature that distinguishes androgen-dependent PCa cells from androgen-independent cells with metabolomic profiles of androgen-treated VCaP cells and prostate derived tissues.
(PDF)

Figure S4 Oncomine concept map (OCM) analysis of 12 metabolite signature concordant between PCa-cell lines and organ-confined tumor/metastatic disease, describe methionine metabolism, methylation and nicotinamide metabolism.
(PDF)

Table S1 List of selected molecular concepts enriched by prostate cancer cell line-specific metabolomic signature.
(PDF)

Table S2 List of all molecular concepts enriched by prostate cancer cell line-specific metabolomic signature.
(PDF)

Table S3 List of all molecular concepts enriched in VCaP cells treated with 10 nM R1881 for 24 h.
(PDF)

Table S4 List of all concepts enriched by 12 metabolite signature that is concordant between PCa cell lines and tissues.
(PDF)
