# Supplementary figures and images for: Metabolomic Profiling Reveals a Role for Androgen in Activating Amino Acid Metabolism and Methylation in Prostate Cancer Cells
Source: PLoS One. 2011 Jul 18;6(7):e21417. doi: 10.1371/journal.pone.0021417 (PMC3138744; doi:10.1371/journal.pone.0021417)

**Figure S1**

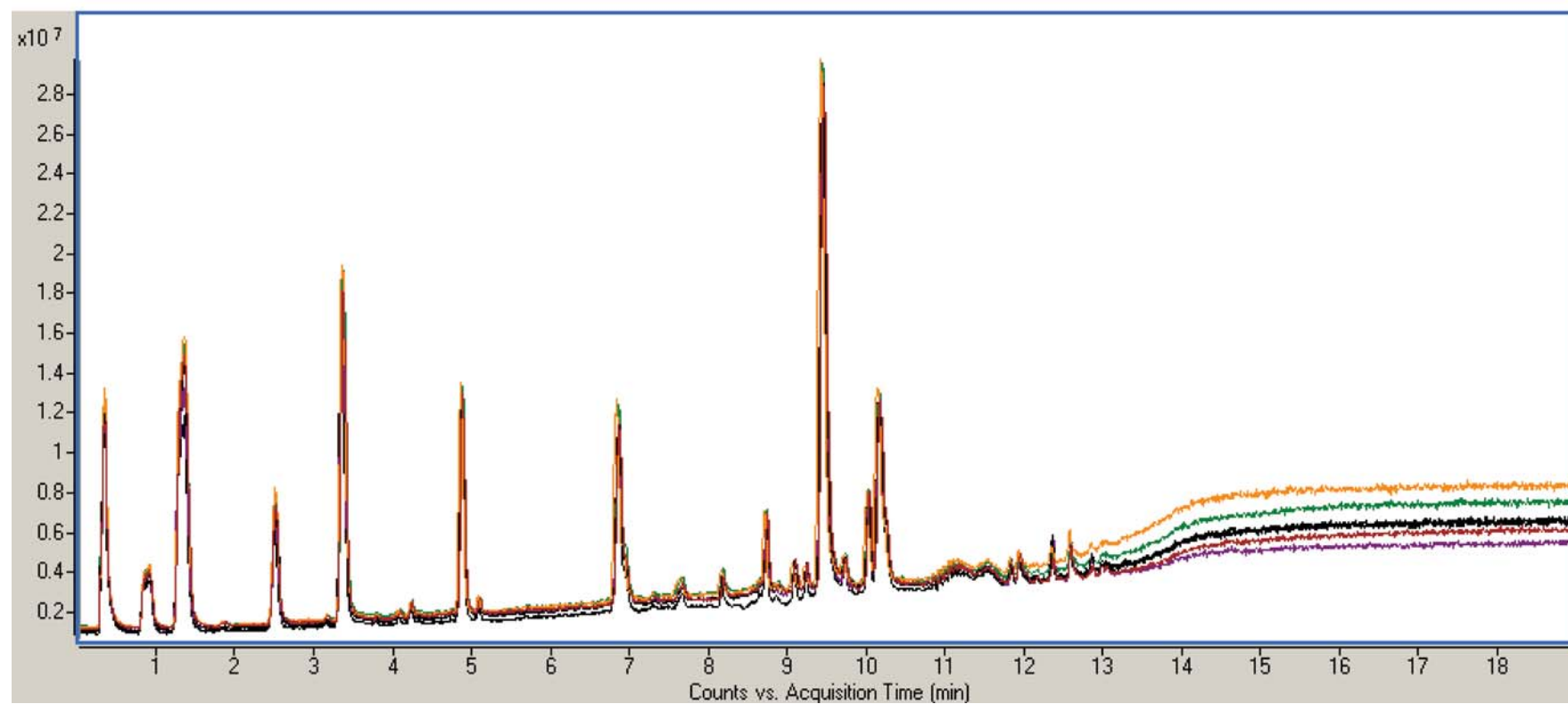

Supplement: Figure S1 — Reproducibility of metabolomic profiling platform used in the discovery phase. Chromatographic reproducibility of a mixture of 12 metabolite standards over five technical replicates anaylyzed on the Q-TOF using positive ionization. (PDF) [file pone.0021417.s001.pdf]

**Figure S2**

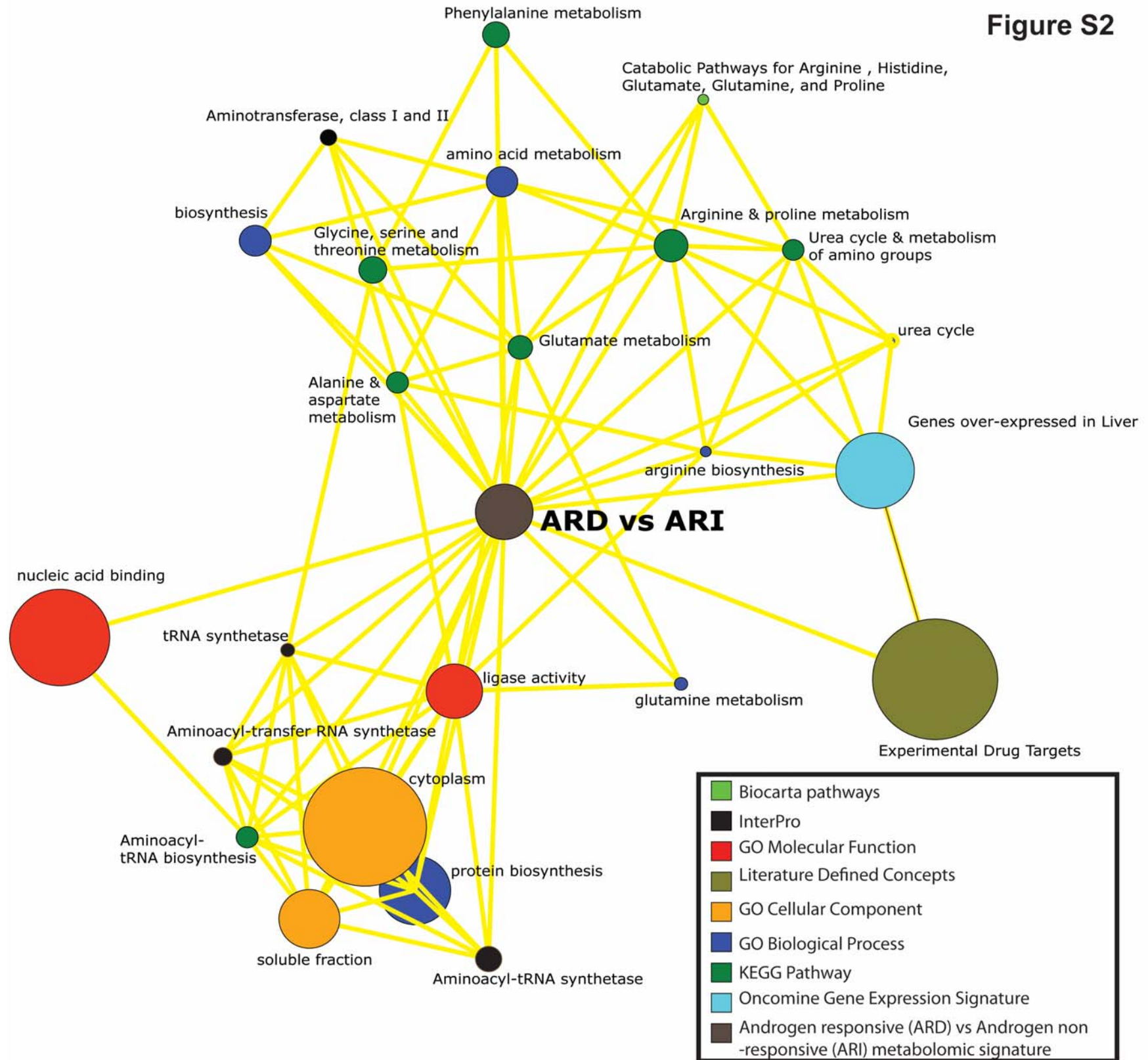

Supplement: Figure S2 — Network view of the molecular concept analysis for the metabolomic profiles of our “metabolic profiles that are altered in ARD vs ARI cell lines” (grey node). Each node represents a molecular concept or a set of biologically related genes. The node size is proportional to the number of genes in the concept. Each edge represents a statistically significant enrichment (FDR q-value<0.2). Enriched concepts describing “amino acid metabolism” are indicated by yellow bridges. (PDF) [file pone.0021417.s002.pdf]
