# Supplementary material for: Metabolomic Profiling Reveals a Role for Androgen in Activating Amino Acid Metabolism and Methylation in Prostate Cancer Cells
Source: PLoS One. 2011 Jul 18;6(7):e21417. doi: 10.1371/journal.pone.0021417 (PMC3138744; doi:10.1371/journal.pone.0021417)
Supplement: Figure S3 — Table describing the concordance of metabolomic signature that distinguishes androgen-dependent PCa cells from androgen-independent cells with metabolomic profiles of androgen-treated VCaP cells and prostate derived tissues. (PDF) [file pone.0021417.s003.pdf]

Figure S3

NO	PCa cell line_ARD vs ANR*	AR24	AR48	NvT	TvM
1	NICOTINAMIDE DINUCLEOTIDE (NAD)	C	C	C	C
2	S-ADENOSYLMETHIONINE (SAM)	C	C	C	C
3	HOMOCYSTEINE	C	C	C	NC
4	CREATININE	C	C	C	NC
5	ASPARAGINE	C	C	NC	C
6	SERINE	C	C	NC	C
7	GLUTAMIC ACID	C	C	NC	NC
8	THREONINE	C	C	NC	NC
9	BETAINE	C	C	ND	ND
10	PHENACETURIC ACID	C	C	ND	ND
11	HISTIDINE	C	NC	C	C
12	TRYPTOPHAN	C	NC	C	C
13	HYPOXANTHINE	C	ND	C	NC
14	CREATINE	C	NC	ND	ND
15	ALANINE	C	ND	NC	NC
16	N1-ACETYLSPERMINE	C	ND	ND	ND
17	ASPARTIC ACID	NC	C	C	NC
18	TYROSINE	NC	C	C	NC
19	LYSINE	NC	C	NC	C
20	XANTHINE	ND	C	NC	NC
21	BENZOIC ACID	NC	C	ND	ND
22	UROCANIC ACID	ND	C	ND	ND
23	GUANINE	NC	C	NC	NC
24	1-METHYL-L-TRYPTOPHAN	ND	C	ND	ND
25	AMINOBUTYRIC ACID	ND	C	ND	ND
26	THYMIDINE	NC	C	ND	ND
27	LEUCINE	NC	NC	C	C
28	OLEIC ACID	ND	ND	C	C
29	PHENYLALANINE	NC	NC	C	C
30	SPERMINE	NC	NC	C	C
31	URACIL	NC	NC	C	C
32	HISTAMINE	ND	ND	C	C
33	KYNURENINE	ND	NC	C	C
34	SPERMIDINE	NC	NC	C	NC
35	ADENINE	ND	NC	C	NC
36	KYNURENIC ACID	ND	NC	NC	C
37	PIPECOLIC ACID	ND	ND	NC	C
38	THYMINE	ND	ND	NC	C
39	ADENOSINE	ND	NC	NC	NC
40	CITRULLINE	NC	ND	NC	NC
41	ARGININE	NC	NC	ND	ND
42	HIPPURIC ACID	ND	ND	NC	NC
43	B-HYDROXYISOVALERIC ACID	NC	NC	ND	ND

Key
C : Concordant
NC: Non-concordant
ND: Not detected
PCa cell line_ARD vs ANR: AR responsive vs non-responsive PCa cell line-derived metabolic signature
AR24: Metabolic signature in VCaP cells treated with 10 nM R1881 for 24h
AR48: Metabolic signature in VCaP cells treated with 10 nM R1881 for 48h
NvT: Metabolic signature in localized PCa
TvM: Metabolic signature in metastatic disease

* Only 43/53 compounds that were detected in atleast one of the four data sets are listed
