# Supplementary material for: Metabolomic Profiling Reveals a Role for Androgen in Activating Amino Acid Metabolism and Methylation in Prostate Cancer Cells
Source: PLoS One. 2011 Jul 18;6(7):e21417. doi: 10.1371/journal.pone.0021417 (PMC3138744; doi:10.1371/journal.pone.0021417)
Supplement: Figure S4 — Oncomine concept map (OCM) analysis of 12 metabolite signature concordant between PCa-cell lines and organ-confined tumor/metastatic disease, describe methionine metabolism, methylation and nicotinamide metabolism. (PDF) [file pone.0021417.s004.pdf]

Figure S4

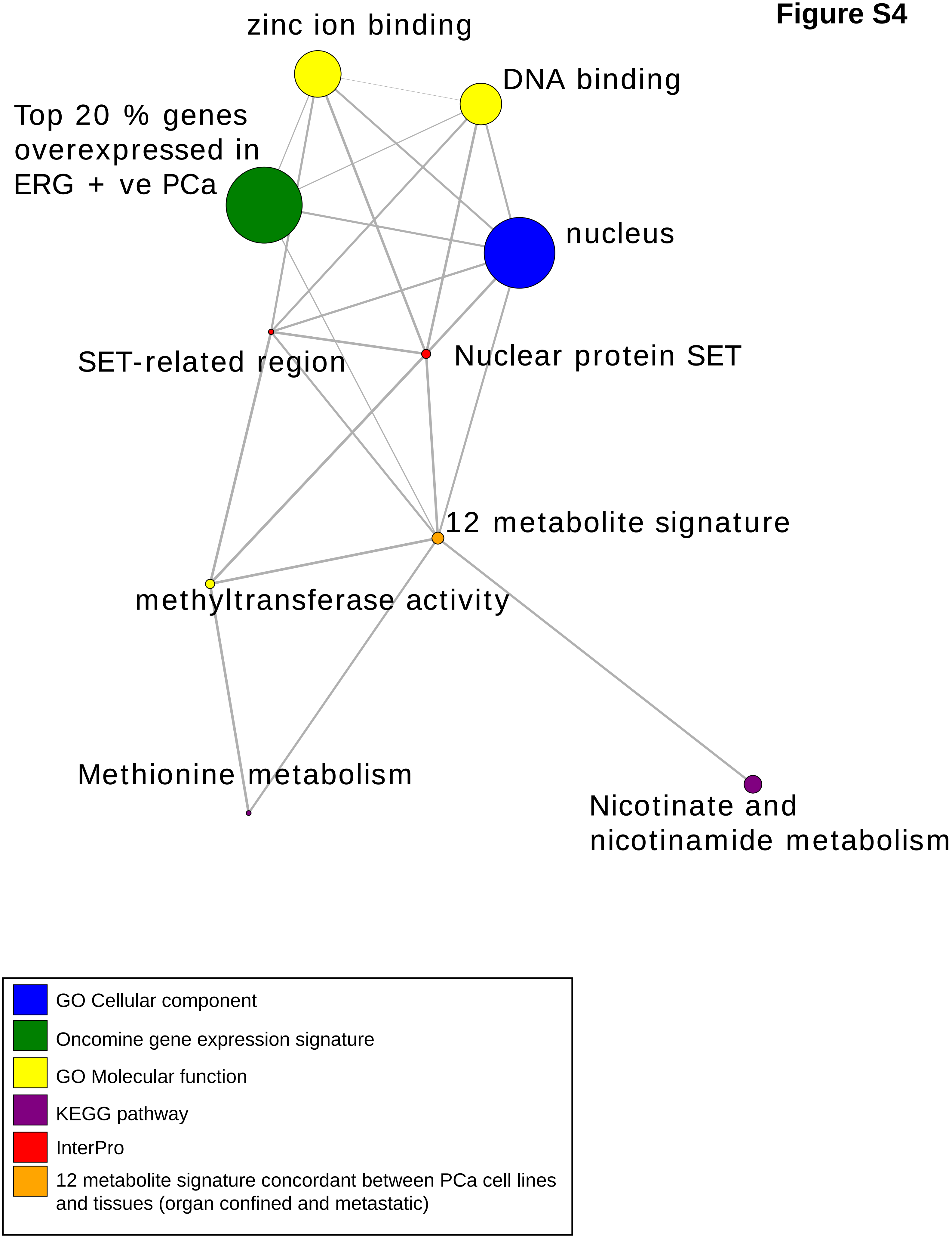

zinc ion binding
DNA binding
Top 20 % genes overexpressed in ERG + ve PCa
nucleus
SET-related region
Nuclear protein SET
12 metabolite signature
methyltransferase activity
Methionine metabolism
Nicotinate and nicotinamide metabolism
GO Cellular component
Oncomine gene expression signature
GO Molecular function
KEGG pathway
InterPro
12 metabolite signature concordant between PCa cell lines and tissues (organ confined and metastatic)
