# Supplementary material for: Metabolomic Profiling Reveals a Role for Androgen in Activating Amino Acid Metabolism and Methylation in Prostate Cancer Cells
Source: PLoS One. 2011 Jul 18;6(7):e21417. doi: 10.1371/journal.pone.0021417 (PMC3138744; doi:10.1371/journal.pone.0021417)
Supplement: Table S1 — List of selected molecular concepts enriched by prostate cancer cell line-specific metabolomic signature. (PDF) [file pone.0021417.s005.pdf]

Table S1

Group Type	Group Name	P-Value	Q-Value
Literature-defined Concepts	Experimental Drug Targets	2.90E-23	1.91E-20
KEGG Pathway	Glutamate metabolism	8.10E-19	6.87E-17
KEGG Pathway	Alanine and aspartate metabolism	5.50E-19	9.27E-17
KEGG Pathway	Arginine and proline metabolism	9.90E-16	5.58E-14
KEGG Pathway	Urea cycle and metabolism of amino groups	1.40E-15	5.75E-14
GO Molecular Function	ligase activity	2.70E-12	5.6E-09
GO Biological Process	amino acid metabolism	2.10E-11	3.82E-08
KEGG Pathway	Aminoacyl-tRNA biosynthesis	1.30E-09	4.49E-08
GO Biological Process	glutamine metabolism	1.30E-10	1.21E-07
Biocarta Pathway	Catabolic Pathways for Arginine , Histidine, Glutamate, Glutamine, and Proline	6.00E-09	1.57E-06
GO Biological Process	protein biosynthesis	4.30E-09	2.66E-06
InterPro	Aminoacyl-transfer RNA synthetase, class II	2.00E-08	9.33E-05
GO Cellular Component	cytoplasm	4.60E-06	0.00201
GO Biological Process	proline biosynthesis	5.70E-06	0.002621
GO Molecular Function	5-nucleotidase activity	5.70E-06	0.005931
GO Molecular Function	transaminase activity	5.70E-06	0.005931
HPRD Interaction Sets	KARS	6.10E-06	0.00665
HPRD Interaction Sets	SCYE1	6.10E-06	0.00665
HPRD Interaction Sets	MARS	6.10E-06	0.00665
HPRD Interaction Sets	IARS	6.10E-06	0.00665
HPRD Interaction Sets	QARS	6.10E-06	0.00665
GO Biological Process	amino acid biosynthesis	1.90E-05	0.006963
InterPro	Aminoacyl-tRNA synthetase, class I	3.20E-06	0.007609
InterPro	Pyridoxal-dependent decarboxylase	5.50E-06	8.63E-03
HPRD Interaction Sets	LARS	2.80E-06	0.015185
HPRD Interaction Sets	EEF1E1	2.80E-06	0.015185
HPRD Interaction Sets	RARS	2.80E-06	0.015185
HPRD Interaction Sets	EPRS	2.80E-06	0.015185
