# Supplementary material for: Metabolomic Profiling Reveals a Role for Androgen in Activating Amino Acid Metabolism and Methylation in Prostate Cancer Cells
Source: PLoS One. 2011 Jul 18;6(7):e21417. doi: 10.1371/journal.pone.0021417 (PMC3138744; doi:10.1371/journal.pone.0021417)
Supplement: Table S2 — List of all molecular concepts enriched by prostate cancer cell line-specific metabolomic signature. (PDF) [file pone.0021417.s006.pdf]

Table S2

Group Type	Group Name	P-Value	Q-Value
Literature-defined Concepts	Experimental Drug Targets	0.000	1.91E-20
KEGG Pathway	Alanine and aspartate metabolism	0.000	9.27E-17
KEGG Pathway	Glutamate metabolism	0.000	6.87E-17
KEGG Pathway	Arginine and proline metabolism	0.000	5.58E-14
KEGG Pathway	Urea cycle and metabolism of amino groups	0.000	5.75E-14
GO Molecular Function	ligase activity	0.000	5.6E-09
GO Biological Process	amino acid metabolism	0.000	3.82E-08
GO Biological Process	glutamine metabolism	0.000	1.21E-07
KEGG Pathway	Aminoacyl-tRNA biosynthesis	0.000	4.49E-08
GO Biological Process	protein biosynthesis	0.000	2.66E-06
Biocarta Pathway	Catabolic Pathways for Arginine , Histidine, Glutamate, Glutamine, and Proline	0.000	1.57E-06
InterPro	Aminoacyl-transfer RNA synthetase, class II	0.000	9.33E-05
HPRD Interaction Sets	RARS	0.000	0.015185
HPRD Interaction Sets	EPRS	0.000	0.015185
HPRD Interaction Sets	EEF1E1	0.000	0.015185
HPRD Interaction Sets	LARS	0.000	0.015185
InterPro	Aminoacyl-tRNA synthetase, class I	0.000	0.007609
GO Cellular Component	cytoplasm	0.000	0.00201
InterPro	Pyridoxal-dependent decarboxylase	0.000	8.63E-03
GO Biological Process	proline biosynthesis	0.000	0.002621
GO Molecular Function	transaminase activity	0.000	0.005931
GO Molecular Function	5-nucleotidase activity	0.000	0.005931
HPRD Interaction Sets	IARS	0.000	0.00665
HPRD Interaction Sets	QARS	0.000	0.00665
HPRD Interaction Sets	MARS	0.000	0.00665
HPRD Interaction Sets	SCYE1	0.000	0.00665
HPRD Interaction Sets	KARS	0.000	0.00665
HPRD Interaction Sets	DARS	0.000	0.006472
Oncomine gene expression	Normal Type - Top 5% over-expressed in Liver (Su)	0.000	0.080861
InterPro	Anticodon-binding	0.000	0.021505
GO Biological Process	amino acid biosynthesis	0.000	0.006963
Literature-defined Concepts	Down-regulated genes in renal cell carcinoma	0.000	0.010441
KEGG Pathway	Glycine, serine and threonine metabolism	0.000	1.08E-03
Oncomine gene expression	Liver Type - Top 10% under-expressed in Metastasis to Liver (Chen)	0.000	7.22E-02
InterPro	tRNA synthetase, class II (G, H, P and S)	0.000	0.04354
Oncomine Clusters	Co-expressed across 64 Lymphoma samples (Rosenwald_Lymphoma_2)	0.000	0.134721
InterPro	Glutamine amidotransferase, class-II	0.000	5.84E-02
InterPro	ATP:guanido phosphotransferase	0.000	5.84E-02
InterPro	Aminotransferase, class V	0.000	5.84E-02
