# Supplementary material for: Metabolomic Profiling Reveals a Role for Androgen in Activating Amino Acid Metabolism and Methylation in Prostate Cancer Cells
Source: PLoS One. 2011 Jul 18;6(7):e21417. doi: 10.1371/journal.pone.0021417 (PMC3138744; doi:10.1371/journal.pone.0021417)
Supplement: Table S3 — List of all molecular concepts enriched in VCaP cells treated with 10 nM R1881 for 24 h. (PDF) [file pone.0021417.s007.pdf]

Table S3

Group Type	Group Name	P-Value	Q-Value
InterPro	Nuclear protein SET	9.00E-24	4.24E-20
KEGG Pathway	Glutamate metabolism	3.60E-19	6.14E-17
GO Molecular Function	methyltransferase activity	2.30E-17	4.8E-14
InterPro	SET-related region	4.30E-14	1.02E-10
GO Biological Process	chromatin modification	6.90E-12	1.27E-08
Literature-defined Concepts	Experimental Drug Targets	8.60E-12	5.68E-09
GO Biological Process	glutamine metabolism	1.10E-10	9.89E-08
GO Molecular Function	histone-lysine N-methyltransferase activity	1.10E-10	1.12E-07
GO Molecular Function	ligase activity	2.50E-09	1.75E-06
GO Biological Process	amino acid metabolism	3.60E-09	2.23E-06
InterPro	Pre-SET	3.20E-08	5.01E-05
HPRD Interaction Sets	HDAC1	3.60E-08	0.000196
KEGG Pathway	Alanine and aspartate metabolism	1.00E-07	8.59E-06
InterPro	Nuclear protein Zn2+-binding	4.50E-07	0.000529
InterPro	Carbamoyl-phosphate synthetase large chain, N-terminal	4.50E-07	0.000529
HPRD Interaction Sets	DNMT3A	5.60E-07	0.001518
Biocarta Pathway	Catabolic Pathways for Arginine , Histidine, Glutamate, Glutamine, and Proline	1.20E-06	0.000306
InterPro	Carbamoyl-phosphate synthase L chain, ATP-binding	1.70E-06	1.33E-03
Oncomine Clusters	Co-expressed across 13 Brain samples (Khatua_Brain)	3.60E-06	0.041212
GO Molecular Function	biotin carboxylase activity	5.10E-06	0.002651
GO Molecular Function	biotin binding	5.10E-06	0.002651
GO Biological Process	DNA methylation	5.10E-06	0.002344
GO Biological Process	NAD biosynthesis	5.10E-06	0.002344
GO Molecular Function	5-nucleotidase activity	5.10E-06	0.002651
InterPro	PWWP	6.10E-06	4.13E-03
InterPro	Rudiment single hybrid motif	6.10E-06	4.13E-03
InterPro	Biotin carboxylase, C-terminal	6.10E-06	4.13E-03
InterPro	AWS	6.10E-06	4.13E-03
InterPro	Biotin-binding site	6.10E-06	4.13E-03
KEGG Pathway	Alkaloid biosynthesis I	9.80E-06	0.000551
HPRD Interaction Sets	DNMT1	1.20E-05	0.022533
HPRD Interaction Sets	DNMT3B	1.20E-05	0.022533
