# Supplementary material for: Metabolomic Profiling Reveals a Role for Androgen in Activating Amino Acid Metabolism and Methylation in Prostate Cancer Cells
Source: PLoS One. 2011 Jul 18;6(7):e21417. doi: 10.1371/journal.pone.0021417 (PMC3138744; doi:10.1371/journal.pone.0021417)
Supplement: Table S4 — List of all concepts enriched by 12 metabolite signature that is concordant between PCa cell lines and tissues. (PDF) [file pone.0021417.s008.pdf]

Table S4

Group Type	Group Name	P-Value	Q-Value
**GO Molecular Function**	**methyltransferase activity**	**1.90E-08**	**3.86515E-05**
**GO Cellular Component**	**nucleus**	**3.90E-07**	**0.000170133**
**InterPro**	**Nuclear protein SET**	**2.20E-07**	**0.001040343**
**KEGG Pathway**	**Nicotinate and nicotinamide metabolism**	**2.30E-05**	**0.003830887**
**KEGG Pathway**	**Methionine metabolism**	**6.50E-05**	**0.005532044**
Conserved UTR Motif	CLUSTER_7__MIR-29C MIR-29B MIR-29A MIR196	6.70E-04	0.047902318
**GO Molecular Function**	**DNA binding**	**1.60E-04**	**0.169526443**
**GO Molecular Function**	**zinc ion binding**	**1.60E-04**	**0.169526443**
**InterPro**	**SET-related region**	**7.80E-05**	**0.184952092**
**Oncomine Gene Expression Signatures**	**Localized Prostate Carcinoma PCa ETS - Top 20% over-expressed in ERG+ (xiaojun)**	**6.50E-05**	**0.363401091**
KEGG Pathway	Purine metabolism	0.01	0.422128373
KEGG Pathway	Selenoamino acid metabolism	0.01	5.41E-01
GO Molecular Function	histone-lysine N-methyltransferase activity	0.001	0.544517978
GO Biological Process	chromatin modification	4.20E-04	0.765878138
Oncomine Gene Expression Signatures	CellLine Vinblastine-newanalysis - Top 20% under-expressed in Indeterminate, Resi	9.00E-04	8.40E-01
Transfac TF Matrix - 1000bp	XFD-3	0.01	0.89845999
Transfac TF Matrix - 1000bp	NF-muE1	0.003	0.927962342
GO Molecular Function	magnesium ion binding	0.002	0.933790176
Transfac TF Matrix - 1000bp	YY1	0.005	0.962902402
GO Molecular Function	transferase activity	0.003	1.01E+00
picTar predicted miRNA target genes	HSA-MIR-29B	0.006	1.045814262
picTar predicted miRNA target genes	HSA-MIR-29C	0.006	1.045814262
Oncomine Gene Expression Signatures	Prostate SPINK1 - Top 10% over-expressed in SPINK1 (LaTulippe)	0.002	1.059810563
Oncomine Gene Expression Signatures	Glioma Grade - Top 5% over-expressed in 4 (Rickman)	0.001	1.08E+00
Oncomine Gene Expression Signatures	Gastric Carcinoma H. Pyroli status - Top 10% under-expressed in positive (Leung)	0.002	1.09E+00
Oncomine Gene Expression Signatures	Parathyroid Type - Top 5% under-expressed in Nonfamilial Multiple Gland Neoplasia	0.002	1.09E+00
Oncomine Gene Expression Signatures	Ovarian Carcinoma Histologic Subtype - Top 10% under-expressed in Mucinous (Schw	0.003	1.10E+00
Oncomine Gene Expression Signatures	Localized Prostate Carcinoma PCa ETS - Top 10% over-expressed in ERG+ (xiaojun)	7.90E-04	1.107844412
Oncomine Gene Expression Signatures	Prostate SPINK1 - Top 5% under-expressed in SPINK1 (Vanaja)	0.002	1.136350429
Transfac TF Matrix - 1000bp	KROX	0.01	1.15E+00
Oncomine Gene Expression Signatures	CellLine Daunorubicin - Top 20% under-expressed in Resistant (Shankavaram)	6.30E-04	1.18E+00
Oncomine Gene Expression Signatures	Parathyroid Type - Top 10% under-expressed in Nonfamilial Multiple Gland Neoplas	0.003	1.180091663
Oncomine Gene Expression Signatures	Ovary Type - Top 5% under-expressed in Ovarian Metastasis to Omentum (Adib)	0.003	1.24E+00
Oncomine Gene Expression Signatures	Brain Type - Top 5% under-expressed in Astrocytoma (Shai)	0.003	1.24E+00
Oncomine Gene Expression Signatures	Bladder Grade - Top 20% over-expressed in High Grade (Dyrskjot)	0.004	1.32E+00
Oncomine Gene Expression Signatures	Germ Cell Type - Top 10% under-expressed in Seminoma (Sperger)	0.004	1.32E+00
Oncomine Gene Expression Signatures	Esophageal Squamous Cell Carcinoma Family history of cancer - Top 10% over-expre	5.00E-04	1.412679118
Oncomine Gene Expression Signatures	Prostate ETS Status - Top 20% over-expressed in ERG+ (xiaojun)	0.006	1.63E+00
Conserved Promoter Motif	CLUSTER_7__V$AP1_C	0.009	1.641197672
Oncomine Gene Expression Signatures	Melanoma Type - Top 20% over-expressed in Lymph Node Metastasis, Metastatic Gr	0.01	1.66E+00
Oncomine Gene Expression Signatures	Cancer Type - v1 - Top 10% over-expressed in Endometrial Endometrioid Carcinoma	0.009	1.664373194
Oncomine Gene Expression Signatures	Cancer Type - v1 - Top 10% over-expressed in Metastatic Ovarian Carcinoma (Bittner	0.009	1.70E+00
Oncomine Gene Expression Signatures	Normal Type - Top 5% over-expressed in Uterus (Shyamsundar)	0.008	1.736146304
Oncomine Gene Expression Signatures	Breast Type - Top 10% under-expressed in Breast Carcinoma (Finak)	0.009	1.75E+00
Oncomine Gene Expression Signatures	Diffuse Large B-cell Lymphoma Subtype - Top 5% over-expressed in Activated B-Cell	0.008	1.76E+00
Oncomine Gene Expression Signatures	Sarcoma Type - Top 10% under-expressed in chondroblastoma (Henderson)	0.009	1.76E+00
Oncomine Gene Expression Signatures	B-cell Chronic Lymphocytic Leukemia Disease Status - Top 10% under-expressed in P	0.007	1.781038862
Oncomine Gene Expression Signatures	Breast Type - Top 5% under-expressed in Breast Carcinoma (Finak)	0.008	1.79E+00
Oncomine Gene Expression Signatures	Acute Myeloid Leukemia Karyotype - Top 10% over-expressed in -7 (Oshima)	0.009	1.80E+00
Oncomine Gene Expression Signatures	Normal Type - Top 10% over-expressed in Testis (Su)	0.009	1.80E+00
Oncomine Gene Expression Signatures	CellLine taxotere-newanalysis - Top 20% under-expressed in Resistant (Staunton)	0.007	1.82E+00
Oncomine Gene Expression Signatures	Bladder Patient status A/D - Top 10% under-expressed in DOD (Modlich)	0.007	1.829243916
Oncomine Gene Expression Signatures	CellLine Daunorubicin - Top 5% under-expressed in Resistant (Shankavaram)	0.007	1.91E+00
Literature-defined Concepts	Genes expressed only in human embryonic stem cells as compared to hematopoieti	0.005	3.395497156
InterPro	Pre-SET	0.007	1.11E+01
Oncomine Clusters	Co-expressed across 16 Breast samples (Weigelt_Breast)	0.008	14.52639303
Oncomine Clusters	Co-expressed across 24 Breast samples (Chang_Breast)	0.007	16.73446596
Connectivity Map	MCF Cells Treatment - Top 5% under-expressed in 506, MCF7 treated with valproic a	0.009	27.62173097
Connectivity Map	CellLine Batch - description - Top 5% under-expressed in 109, SKMEL5 treated with n	0.009	27.62173097
